# Interpersonal Contributors to Depression in Sexual Minority Adolescents: An Examination of Exposure to Acute Stressors and Neural Reactivity to Interpersonal Emotional Images

**DOI:** 10.1002/dev.70092

**Published:** 2025-12-02

**Authors:** Yinru Long, Corinne N. Carlton, Samantha Pegg, Lisa Venanzi, Sarah E. Woronko, Kirsty A. Clark, Autumn Kujawa

**Affiliations:** ^1^ Department of Psychology and Human Development Vanderbilt University Nashville Tennessee USA; ^2^ Department of Medicine, Health, and Society Vanderbilt University Nashville Tennessee USA

**Keywords:** adolescents, depression, electroencephalogram (EEG), emotional reactivity, interpersonal stress, sexual orientation

## Abstract

Sexual minority (SM) adolescents are at a higher risk of depression compared to their heterosexual peers, indicating a need for multimethod research to identify risk and resilience factors for SM youth. In a sample of 165 adolescents who completed an interview‐based acute stress exposure assessment and an interpersonal emotional images task to elicit the late positive potential (LPP) derived from electroencephalogram (EEG), we aimed to investigate (1) differences in the severity of acute interpersonal stress exposure (including peer and family stressors) and depressive symptoms in SM relative to heterosexual adolescents, (2) neural responses to interpersonal emotional images as moderators of the association between SM identity and depressive symptoms, and (3) whether these interactions persist when accounting for disparities in exposure to recent interpersonal stressors. SM adolescents reported higher depressive symptoms and were exposed to more severe recent peer (but not family) stressors than heterosexual adolescents. LPP responses to both positive and negative interpersonal images moderated the association between SM identity and depressive symptoms, such that a relatively blunted LPP potentiated the association. Interactions remained significant when accounting for the severity of peer stressors, suggesting the unique role of neural responses to interpersonal emotional images in depressive symptoms in SM adolescents.

## 1 Introduction

Sexual minority (SM) adolescents, which broadly include adolescents with any nonheterosexual sexual orientation (e.g., gay, bisexual) or who report same‐gender romantic or sexual attraction, face a heightened risk of depression. Substantial evidence shows that rates of depressive disorders and severity of depressive symptoms are higher in SM adolescents as compared to their heterosexual peers (Lucassen et al. 2017; Marshal et al. 2011). A recent national study using data from the Youth Risk Behavior Surveillance System found that SM adolescents were more than twice as likely to endorse depressive symptoms as their heterosexual peers (Depa et al. 2022). Heightened exposure to interpersonal stressors, including peer bullying and family rejection, is thought to be a key factor underlying this disparity (Goldbach and Gibbs 2017; Russell et al. 2011; Russell and Fish 2019). The framework proposed by minority stress theory lays out an array of psychological and social stressors related to SM identity, including external minority stressors such as bullying and discrimination, as well as internal minority stressors such as internalized stigmatization and emotional distress related to acceptance (I. H. Meyer 2003; Rosario et al. 2002). These external and internal minority stress experiences can then create elevations in emotional, psychological, and interpersonal risk factors for psychopathology (Hatzenbuehler 2009). They can also impact emotional processes in ways that might contribute to depression disparities, particularly during this developmentally sensitive period when emotion‐related brain function is changing (Allen and Sheeber 2008; Eckstrand, Lenniger, et al. 2022; Forbes et al. 2021; Goldbach and Gibbs 2017). Thus, there is a need to use multiple methods to identify vulnerability factors that shape depression risk in SM adolescents, in combination with these interpersonal stressors.

SM adolescents often encounter interpersonal stressors within two compulsory social environments, including home and school (Goldbach and Gibbs 2017). For example, SM adolescents often report negative family experiences, including conflict with family members and parental rejection or nonacceptance, particularly within the context of sexual orientation identity disclosure (Clark et al. 2022; Puckett et al. 2015; Willoughby et al. 2008), as well as heightened exposure to peer victimization at school (Russell et al. 2011). These negative interpersonal experiences can then foster psychological internalization of these stigmatizing experiences (e.g., rejection sensitivity) (Goldbach and Gibbs 2017; Hatzenbuehler 2009; I. H. Meyer 2003; Singh et al. 2023) and contribute to the sexual orientation disparity in depression during adolescence (Goldbach and Gibbs 2017; la Roi et al. 2016; Pachankis and Clark 2025). A recent systematic review highlighted a significant gap in understanding the neural correlates of minority stress (Nicholson et al. 2022), emphasizing the need to examine individual differences in emotional and neural processes associated with interpersonal experiences that are salient in the lives of SM adolescents (e.g., peer and family‐related experiences). These processes might play a key role in exacerbating or mitigating the risk of depression. Although in early stages, this line of research has the potential to contribute to the understanding of the risk and resilience factors underlying the sexual orientation disparity in depression by accounting for individual processes at the neural level, informing future development of more targeted interventions (Eckstrand, Lenniger, et al. 2022; Forbes et al. 2021)

Adolescence represents a crucial developmental phase characterized by important changes in emotion‐related brain function (Allen and Sheeber 2008), which may play a role in depression disparities for SM adolescents (Singh et al. 2023). Brain functions contributing to emotional processing may be moderating factors that potentiate or attenuate the association between the minority stress experiences of SM adolescents and depressive symptoms (Eckstrand, Lenniger, et al. 2022; Eckstrand, Silk, et al. 2022; Forbes et al. 2021). However, to date, few studies have evaluated this possibility. A few studies using functional magnetic resonance imaging (fMRI) have examined individual differences in brain function that contribute to SM youth mental health and stress responses. For example, in one study, reduced activation in the right temporoparietal junction (TPJ) in response to social reward was associated with interpersonal depressive symptoms, particularly among SM adolescents (Eckstrand et al. 2019). In another study, reduced neural response to social reward in the ventral striatum (VS) was associated with a stronger association between minority victimization and sleep disturbance, which in turn predicted more severe suicidal ideation (Seah et al. 2025). Social reward responsiveness can also be measured through the reward positivity (RewP) derived from event‐related potentials (electroencephalogram [EEG]). In a prior study with the current sample, we found that SM adolescents with relatively blunted RewP to social reward reported higher levels of depressive symptoms and likelihood to endorse suicidal ideation (Long et al. 2024). Affective neuroscience research on SM youth mental health to date has primarily focused on the reward‐related brain process, but extension to neural responses to negative social experiences that are more commonly encountered by SM adolescents is needed.

While reductions in late positive potential (LPP) to both positive and negative images align with the emotion context insensitivity (ECI) theory that characterizes depression by blunted reactivity across both positive and negative emotional contexts (Bylsma 2021), the direction of associations between depressive symptoms and LPP to negative emotional images appears to be more mixed than to positive images. Some studies have shown that depressed people show reduced LPP to threatening faces (Foti et al. 2010), while other evidence also showed associations between heightened emotional reactivity to negative stimuli and depression (Herres et al., 2015). Despite emerging evidence showing the role of individual differences in LPP in responses to stress and depressive symptoms, LPP has yet to be evaluated in the context of depressive symptoms in SM adolescents, who are more likely than their heterosexual peers to be exposed to heightened levels of interpersonal stress (Goldbach and Gibbs 2017).

The present study is among the first to examine both psychosocial and neurophysiological factors in SM youth and associations with depressive symptoms, integrating validated interviewer‐derived measures of stress with an EEG assessment of neural responses to interpersonal emotional images. First, we examined differences in depressive symptoms and severity of recent acute interpersonal stress exposure (with peers and family) between SM and heterosexual adolescents. Next, we evaluated the moderating role of LPP to interpersonal emotional images in the association between SM identity and depressive symptoms. We hypothesized that LPP to positive interpersonal images would moderate the relationship between SM identity and depressive symptoms, such that SM adolescents with a reduced LPP to positive stimuli would report higher levels of depressive symptoms. For LPP to negative images, our hypothesis regarding the direction of associations with depressive symptoms was more exploratory. From the ECI perspective, depression may be characterized by blunted responses to both positive and negative emotional changes in the environment (Bylsma et al. 2008). However, since the negative stimuli were interpersonally threatening, hyperreactivity to these situations may potentiate the relationship between SM identity and depressive symptoms, given increased exposure to interpersonal stressors. Lastly, we tested whether interactions between SM identity and neural processing of emotional images on depressive symptoms persist when accounting for recent exposure to acute interpersonal stress to determine the extent to which stress exposure accounts for these effects.

## 2 Method

### 2.1 Participants

Participants included 165 adolescents aged 14–17 years (*M* = 15.23, *SD* = 1.07); 61.8% identified as female assigned sex at birth. Participants were recruited from advertisements across the local university, academic medical center, and community mental health and pediatrics clinics. We oversampled adolescents for current depression using the Kiddie Schedule for Affective Disorder (K‐SADS) interview (Kaufman and Schweder 2004) to ensure variability in current depressive symptoms (58 participants were in a current depressive episode when enrolled in the study).

With regard to sexual orientation, 76.4% of participants identified as heterosexual, and we categorized the remaining 23.6% of participants who did not identify as heterosexual as SM adolescents (*n* = 37). Among the SM adolescents, three identified as gay, five preferred not to say, seven were not sure, seven preferred to self‐describe (six identified as pansexual and one as lesbian), and 15 as bisexual.^1^ Concerning gender diversity, one participant identified as transgender, and two participants self‐described their gender identities as gender fluid or as using they/them pronouns. All gender minority participants also identified as SM. In terms of race and ethnicity, 69.7% identified as White, 15.8% as Black and/or African American, 6.1% as Asian, 1.2% as American Indian or Alaska Native, 1.2% as Native Hawaiian or Pacific Islander, 6.7% as Hispanic and/or Latinx, and 6% as other.

We previously published results of the interpersonal emotional images task in terms of associations between trauma and depressive symptoms (Long et al. 2023) and the interaction between SM identity and social reward responsiveness on depressive symptoms and suicidal ideation in this same sample (Long et al. 2024), but this study is the first to examine the severity of acute stressors, LPP, and SM identity. A subset of the depressed sample (*n* = 19) also enrolled in another study of depression treatment (Dickey et al. 2023), but measures for the current study were completed prior to beginning treatment.

### 2.2 Procedures

All study procedures were reviewed and approved by the Review Board at Vanderbilt University. Before study procedures began, informed consent was obtained from all parents and informed assent from participants. Postdoctoral researchers and graduate students administered clinical interviews to determine stressful events in the past 6 months (see Section 2.3). Then, participants completed a battery of self‐report questionnaires, including demographics and depressive symptoms, using the online survey platform REDCap (Harris et al. 2019). Finally, participants completed EEG assessments, including the interpersonal emotion images task designed to assess emotional reactivity (described in detail below).

### 2.3 Measures

#### 2.3.1 Severity of Interpersonal Stressors

Clinical postdoctoral researchers and graduate students administered the UCLA Life Stress Interview (Adrian and Hammen 1993) to characterize exposure to acute stressors across several life domains in the past 6 months. The interview has been validated in children and adolescents (Rudolph and Hammen 1999) and involves interviewers probing discrete acute stressful events that happened in the past 6 months using example questions such as “What happened?” or “Was that expected?”. Further probing was used to explore the desirability of the event (e.g., breaking up with someone because they wanted to break up with the person vs. being broken up with might have different ratings), tangible resource availability, coping abilities, and contextual information. In this study, we focused on acute stressors in the peer (e.g., losing friends, being excluded from a friend group) and family (e.g., changes in parent's job, major arguments with family members, having to move from one parent's home to another) domains. Interviewers then prepared an objective summary of the event (i.e., removing all subjective information like participant emotional responses) and then presented these summaries to raters who did not directly interact with the participant for independent coding. Two independent raters each rated 227 events for negative impact, and the intraclass correlation was 0.92. Consistent with prior work (Hammen 1991), events scored 1 (minimal to no impact) were excluded when calculating the sum of negative impact ratings across events. Among the 155 participants who completed the clinical interviews on stressful events, 47.1% endorsed one or more stressful events in the peer domain, and 42.6% endorsed one or more stressful events in the family domain.

#### 2.3.2 Depressive Symptoms

Participants completed the 33‐item Mood and Feeling Questionnaire (MFQ), a self‐report measure of children and adolescents’ depressive symptoms (Angold et al. 1995; Costello and Angold 1988). Sample items include “I felt miserable or unhappy” and “I didn't enjoy anything at all.” Each item was rated on a scale of 0 (*Not true*), 1 (*Sometimes true*), and 2 (*True*). We calculated a mean composite score for the overall depression by summing all 33 items (*M* = 15.96, *SD* = 14.28). The measure has been validated in clinical and nonclinical adolescent samples with excellent reliability (Burleson Daviss et al. 2006). In the current study, Cronbach's alpha was high (α = 0.96).

#### 2.3.3 Interpersonal Emotional Images Task

**FIGURE 1:**
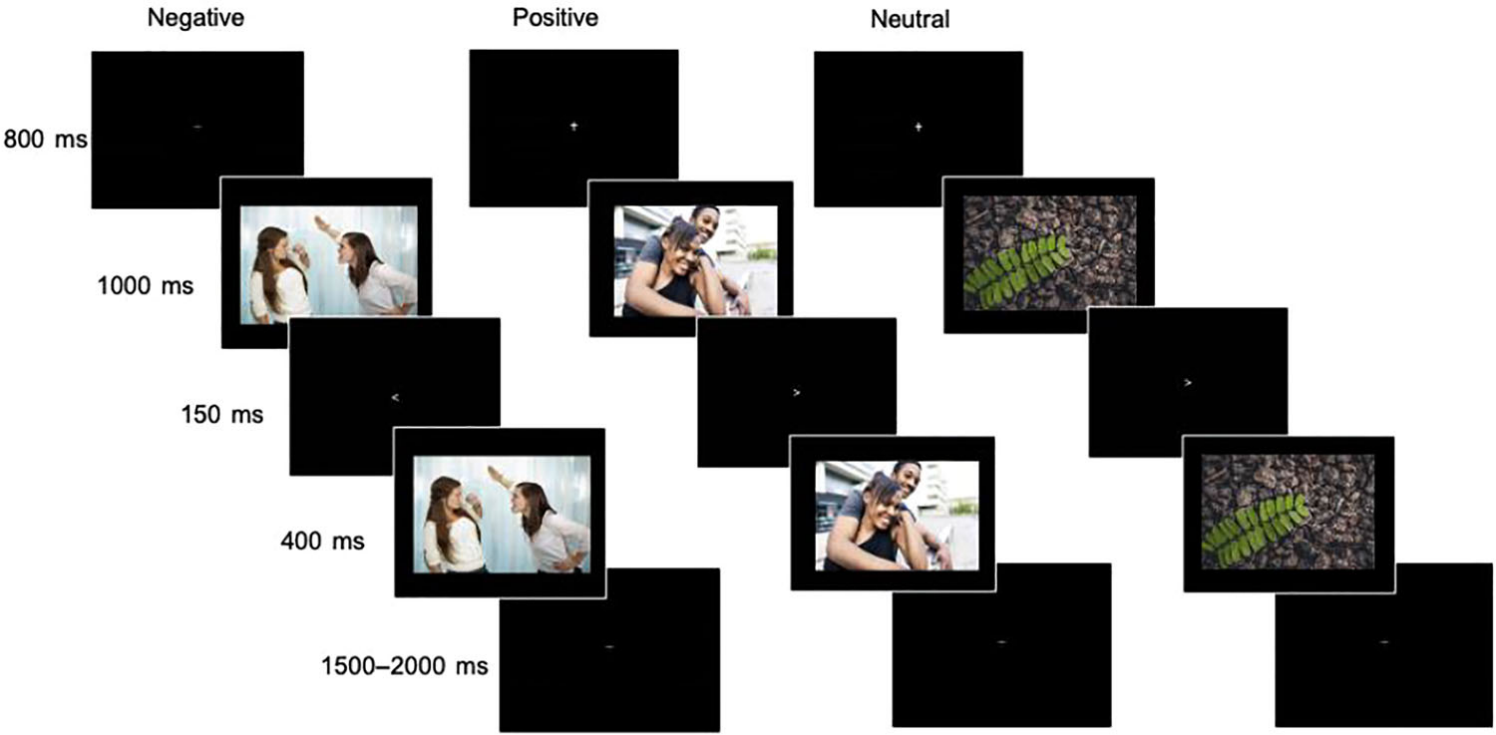
Interpersonal emotional images task.

The interpersonal emotional image task was developed based on an EEG emotional interrupt task (Mitchell et al. 2006; Weinberg and Hajcak 2011) and modified to elicit neural responsiveness to 15 adolescent‐focused interpersonally positive images (e.g., happy parents playing with child, friends hugging or laughing together), 15 interpersonally negative images (e.g., peer bullying, teens and parents arguing), and 15 neutral images (e.g., city scenes, plants, rock) (see Figure 1). This task has also been used in young adults (Dickey, West, et al. 2021), and we previously presented results with this sample of adolescents examining lifetime trauma exposure (Long et al. 2023), though not in relation to sexual orientation or acute stress. Stimuli were primarily obtained through stock image search, using search terms that focused on positive and negative interpersonal events. Three neutral images were derived from the Open Affective Standardized Image Set (OASIS; Kurdi et al. 2016). In total, eighty images were initially selected based on their face validity. Forty‐five images (15 images of each valence) were selected based on face validity, and they showed differences in valence and arousal ratings as expected in emerging adults and the current adolescent sample (Dickey, West, et al. 2021; Long et al. 2023).

Participants completed six practice trials before the task to ensure their understanding. Each trial began with a fixation cross presented for 800 ms, followed by an image for 1000 ms. Next, an arrow pointing either left or right is presented on the screen for 150 ms, and the participants were instructed to indicate the direction of the arrow as an attention check and behavioral measure of accuracy and reaction time. Then, the same pictorial stimuli were present for another 400 ms. Finally, the fixation cross was presented for 1500–2000 ms before the start of the subsequent trial. The task included 90 trials total. Participants completed ratings of valence and arousal of the images after the task. They rated negative images as more unhappy than neutral (*t*(137) = 25.42, *p* < 0.001) and positive images (*t*(137) = 26.81, *p* < 0.001), while positive images were rated as happier than neutral images (*t*(137) = −13.68, *p* < 0.001). Positive (*t*(177) = −8.12, *p* < 0.001, Cohen's *d* = −0.61) and negative (*t*(177) = −6.51, *p* < 0.001, Cohen's *d* = −0.49) stimuli were both rated as more arousing than the neutral stimuli (*p*s < 0.05).

### 2.4 EEG Data Collection and Processing

**FIGURE 2:**
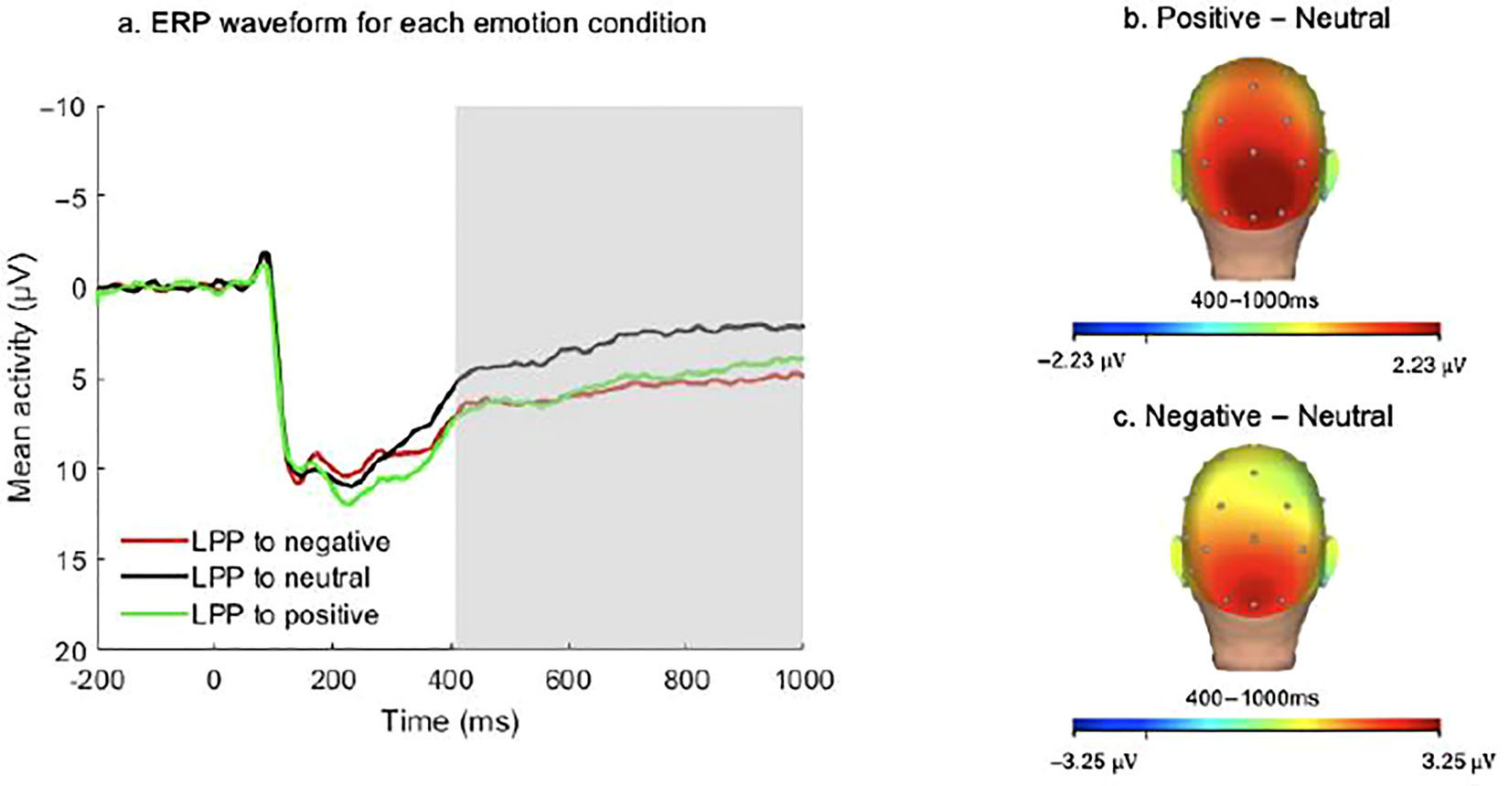
The grand average ERP waveform and the scalp distribution. (a) Grand average ERP waveforms of LPP to negative, neutral, and positive stimuli (negative plotted up) pooled across O1, O2, Oz, and Pz. (b) Scalp distribution of the difference between responses to interpersonally positive versus neutral images 400–1000 ms after stimulus onset. (c) Scalp distribution of the difference between responses to interpersonally negative versus neutral images 400–1000 ms after stimulus onset.

EEG data were continuously recorded using a 32‐channel actiCHamp system from BrainProducts (Munich, Germany). We attached facial electrodes about 1 cm above and below the right eye (VEO) and 1 cm to the side of each eye (HEO) to record electrooculogram (EOG) data for ocular correction later. Based on BrainProducts bipolar‐to‐auxiliary adapter design, a reference electrode was placed on the back of the neck, approximately two fingers’ width above the neck bump. Since our study was conducted throughout the COVID‐19 pandemic, we used a 16‐channel montage for 23 participants recruited during the pandemic to reduce contact (Pegg et al. 2024; Simmons and Luck 2020). We also did not attach EOG electrodes to reduce time exposure in close contact and airborne virus transmission during the capping procedure and used alternative methods to conduct ocular corrections.^2^ Using Brain Vision Analyzer software to process data, we first used a bandpass filter of 0.01–30 Hz to filter out noise. Filtered data were re‐referenced to the recordings of the mastoid electrodes (TP9/TP10) and then segmented into trials from −200 to 1000 ms after image onset. Ocular correction was conducted using VEO and HEO when they were not flat or had not incurred extreme noise. When VEO was unavailable, we used cap electrode FP1 to measure vertical eye moments with common reference; when HEO was not available, we used cap electrode FT9 with FT10 as reference to measure horizontal eye movements (Pegg et al. 2024). Semiautomatic artifact rejection was implemented using the following criteria: maximal allowed voltage step was 50 µV/ms; maximal allowed absolute difference was 175 µV with an interval length of 400 ms; and lowest allowed activity was 0.5 µV with an interval length of 100 ms. The remaining artifacts were removed using visual inspection, and any electrode that exceeded 75% of trials rejected was interpolated before artifact rejection. Segments were averaged for each condition. A baseline correction of −200 to 0 ms prior to stimulus onset was applied to averaged segments within each condition. LPP was scored as mean amplitude from 400 to 1000 ms post‐stimulus and pooled across occipital–parietal sites (O1, O2, Oz, Pz),^3^ consistent with prior research (Dickey, West, et al. 2021; Long et al. 2023) and corresponding to where LPP was maximal in the overall sample (see Figure 2). Split‐half reliability was 0.83 for LPP to positive interpersonal stimuli, 0.80 for LPP to negative interpersonal stimuli, and 0.82 for LPP to neutral stimuli. The ERP waveforms and scalp distributions in the full sample are presented in Figure 2.

### 2.5 Data Analysis

Data were analyzed using *lavaan* package in R (Rosseel 2012). We calculated the unstandardized residuals for LPP to positive or negative emotional stimuli, respectively, when accounting for LPP to neutral stimuli (A. Meyer et al. 2017). Then, to explore Aim 1, we examined the bivariate correlations between all variables of interest. Next, to explore Aim 2, we conducted multiple regression analyses with SM identity and LPPs to positive or negative emotional stimuli as the main predictors, age and sex assigned at birth as covariates, and depressive symptoms as the outcome variable. We also included the interaction terms between LPP residuals and SM identity by taking the product of LPP residuals to emotional stimuli and SM identity. To interpret significant interactions, we generated the interaction plot with LPP to emotional stimuli at the high (1 *SD* above the mean), mean, and low (1 *SD* below the mean) levels. We also generated the simple slopes analyses output and the Johnson–Neyman region of significance using an online computational tool for MLR two‐way interaction (Preacher et al. 2006). Lastly, we repeated analyses covarying for the severity of interpersonal stressors to which SM adolescents were significantly exposed in the model, in order to examine whether interaction effects persist when accounting for disparities in recent stress exposure.

To account for missing data, we used full information maximum likelihood (FIML) with the *lavaan* package in R, which allowed us to use all 165 participants in the model fit (Enders and Bandalos 2001). In our study, eight of the 165 participants did not report sexual orientation, and as such, they were not categorized into either SM or non‐SM groups. There were also six participants missing self‐report data on depressive symptoms due to incomplete questionnaires, and 10 participants did not complete the Life Stress Interview. As for LPPs to interpersonally positive or negative stimuli, 27 participants were missing EEG data (13 participants did not complete the task, and 14 participants with noisy data were excluded). Grubb's test was conducted with the GraphPad QuickCalcs online calculator (Version 6.0, GraphPad Software) to identify any significant outliers in LPP residuals. We excluded one significant outlier on LPP residuals to negative interpersonal stimuli (*p* < 0.05). To further probe the missing data pattern, we created a dummy‐coded missingness variable for each variable of interest. We conducted bivariate correlations to test if the missingness of any variable was significantly correlated with any demographic or clinical variables. The results revealed that the level of depressive symptoms was positively correlated with whether LPP residuals to emotional stimuli were missing (*r*s = 0.18, *p*s < 0.05), and LPP residuals to both emotional stimuli were positively correlated with whether interview data were missing (*r*s > 0.25, *p*s < 01). Lastly, we also tested for multicollinearity and addressed the skewness of our dependent variable using robust standard errors in Supporting Information S1.

## 3 Results

### 3.1 Descriptive Statistics and Bivariate Correlations

We first conducted bivariate correlations among all variables of interest, including SM identity, severity of peer and family stressors, LPP to emotional stimuli, and depressive symptoms (Table 1). Correlations between two continuous variables were reported as Pearson's *r*; correlations between a dichotomous and a continuous variable were reported as point biserial coefficient (*r*_pb_); and the association between two dichotomous variables was assessed using the chi‐square test and reported as *phi* coefficient. Compared with non‐SM adolescents, SM adolescents reported significantly higher levels of depressive symptoms and were exposed to higher severity of peer stressors but not family stressors. SM and non‐SM adolescents did not significantly differ in LPP responses to positive or negative images. The severity of both peer and family stressors was positively correlated with depressive symptoms.

**TABLE 1:** Bivariate correlations between variables of interest (*N* = 165).

	Variable	Mean (*SD*)/percentage	1	2	3	4	5	6	7	8
1	SM identity	22% SM	—							
2	Age	15.23 (1.07)	0.06	—						
3	Sex	62% female	2.92	−0.04	—					
4	Severity of peer stressors	1.56 (2.27)	0.27^**^	−0.06	0.11	—				
5	Severity of family stressors	1.44 (2.32)	0.06	0.05	0.02	−0.01	—			
6	LPP residuals to negative stimuli	0.00 (3.73)	−0.05	−0.10	−0.14	−0.09	0.12	—		
7	LPP residuals to positive stimuli	0.00 (3.39)	−0.06	−0.05	−0.09	−0.09	0.11	0.58^**^	—	
8	Depressive symptoms	15.96 (14.29)	0.49^**^	0.12	0.10	0.30	0.25	−0.05	−0.03	—

*Note:* Sex assigned at birth coded as 0 = male, 1 = female. Correlations between two binary variables are *φ* coefficients; correlations between two continuous variables are Pearson's *r*; correlations between a dichotomous variable and a continuous variables are point biserial correlation coefficients (*rpb*). FIML was used to account for missing data.

Abbreviations: LPP, late positive potential; SM, sexual minority.

*p < 0.05; **p < 0.01; ***p < 0.001.

### 3.2 Regression Analyses

**FIGURE 3:**
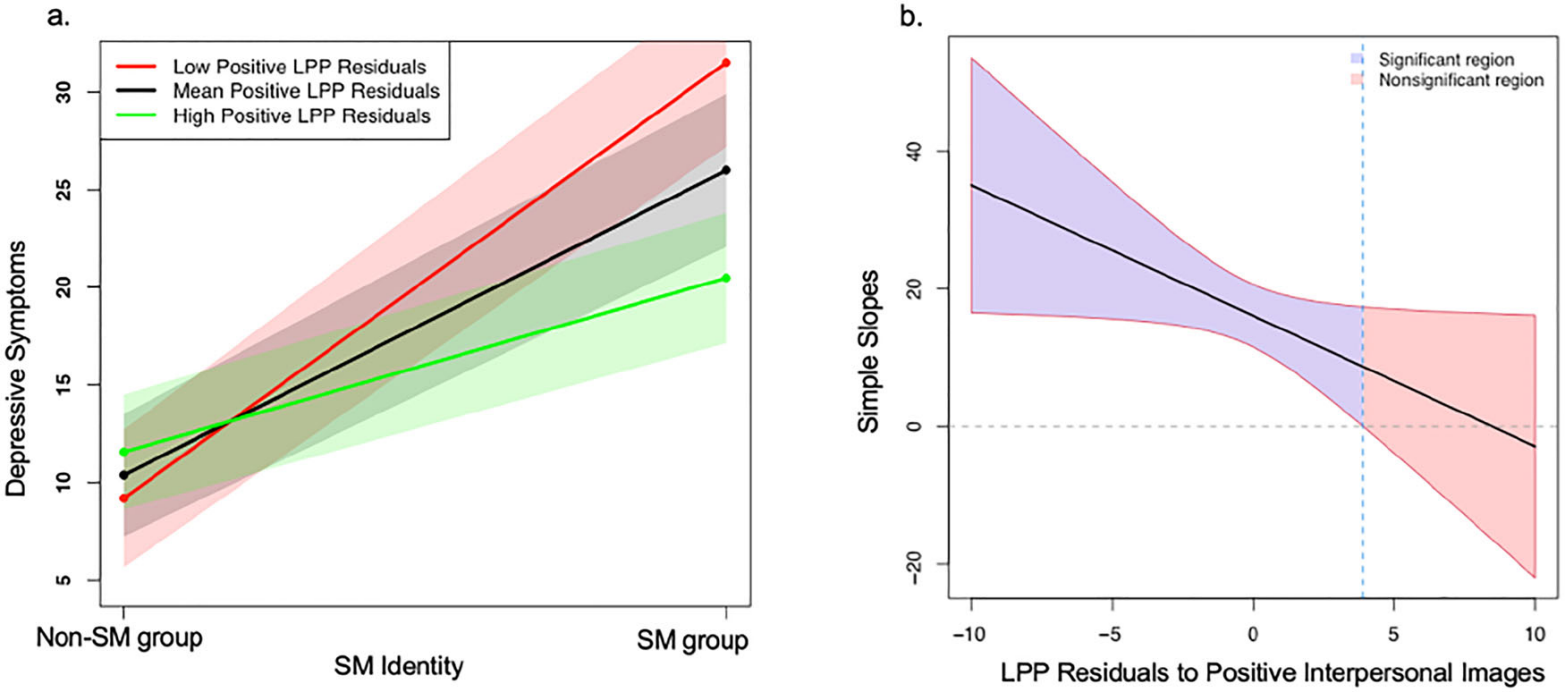
Interaction plot and J–N output of LPP to positive stimuli. (a) Simple slopes analysis of the association between SM identity and depressive symptoms at the low (1 *SD* below the mean), mean, and high (1 *SD* above the mean) levels of LPP residuals to positive stimuli. (b) Slope (with confidence bands and Johnson–Neyman region of significance) of the association between SM identity and depressive symptoms as a function of LPP to positive stimuli.

Multiple regression results showed that LPP to positive interpersonal images significantly moderated the association between SM identity and depressive symptoms (see Table 2). Simple slope analyses indicated that the association between SM identity and depressive symptoms was significant at all levels of LPP to positive stimuli (*p*s < 0.05). However, the association was the weakest at the high (1 *SD* above the mean) level of LPP to positive stimuli (*b* = 9.09, *SE* = 4.02, *t* = 2.26, *p* = 0.03), was somewhat stronger at the mean level of LPP to positive stimuli (*b* = 15.77, *SE* = 2.79, *t* = 5.65, *p* = 0.00), and was the strongest at the low (1 *SD* below the mean) level of LPP to positive stimuli (*b* = 22.46, *SE* = 4.57, *t* = 4.91, *p* = 0.00; Figure 3a). The Johnson–Neyman test (Figure 3ba) indicated that the association between SM identity and depressive symptoms was only significant when LPP to positive stimuli was below 3.55 µV (*M* = 0.00, range = [−6.92 to 10.86]). This interaction remained significant when covarying the severity of peer stressors (*β* = −0.20, *z* = −2.44, *p* = 0.02, *R* ^2^ = 0.32) and when covarying the interaction between the severity of peer stressors and LPP to positive stimuli (*β* = −0.25, *z* = −2.78, *p* = 0.01, *R* ^2^ = 0.32). The interaction between the severity of peer stressors and LPP to positive stimuli on depressive symptom was not significant in the model including the SM × LPP interaction (*β* = 0.13, *z* = 1.32, *p* = 0.19, *R* ^2^ = 0.32).

**TABLE 2:** Multiple regression analyses testing the main and interactive effects of SM identity and LPP residuals to positive interpersonal images on depressive symptoms.

Variable	*β*	*b*	*SE*	*p*	*z*
SM identity	0.47	15.62	2.33	0.00	6.71
LPP residuals to positive stimuli	0.08	0.36	0.37	0.33	0.97
SM identity × LPP residuals to positive stimuli	−0.19	−2.04	0.90	0.02	−2.25

*Note: R* ^2^ = 0.28. FIML was used to account for missing data.

Abbreviations: LPP, late positive potential; SM, sexual minority.

**FIGURE 4:**
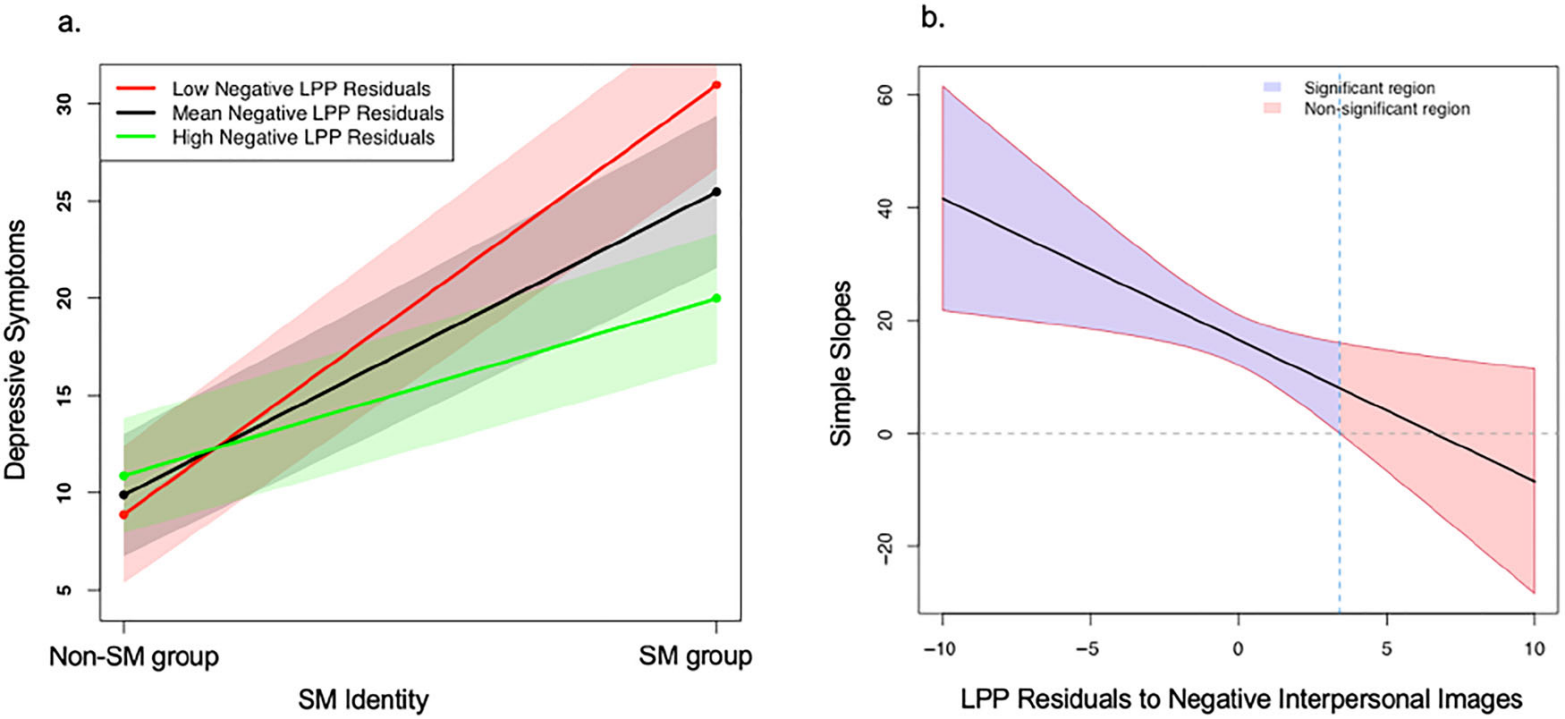
Interaction plot and J–N output for LPP to negative stimuli. (a) Simple slopes analysis of the association between SM identity and depressive symptoms at the low (1 *SD* below the mean), mean, and high (1 *SD* above the mean) levels of LPP residuals to negative stimuli. (b) Slope (with confidence bands and Johnson–Neyman region of significance) of the association between SM identity and depressive symptoms as a function of LPP to negative stimuli.

LPP to negative interpersonal images also moderated the association between SM identity and depressive symptoms (see Table 3). Simple slope analyses indicated that the association between SM identity and depressive symptoms was significant at all levels of LPP to negative stimuli (*p*s < 0.05). However, the association was the weakest at the high (1 *SD* above the mean) level of LPP to negative stimuli (*b* = 9.32, *SE* = 3.86, *t* = 2.42, *p* = 0.02), was somewhat stronger at the mean level of LPP to negative stimuli (*b* = 15.81, *SE* = 2.32, *t* = 6.82, *p* = 0.00), and was the strongest at the low (1 *SD* below the mean) level of LPP to negative stimuli (*b* = 22.30, *SE* = 3.53, *t* = 6.32, *p* = 0.00; Figure 4a). The Johnson–Neyman test (Figure 4b) indicated that the association between SM identity and depressive symptoms was only significant when LPP to negative stimuli was below 4.18 µV (*M* = 0.00, range = [−11.26 to 12.13]). This interaction also remained significant when covarying the severity of peer stressors (*β* = −0.19, *z* = −2.22, *p* = 0.03, *R* ^2^ = 0.31), as well as covarying the interaction between the severity of peer stressors and LPP to negative stimuli (*β* = −0.20, *z* = −2.21, *p* = 0.03, *R* ^2^ = 0.31). The interaction between the severity of peer stressors and LPP to negative stimuli on depressive symptom was also not significant in the model including the SM × LPP interaction (*β* = 0.03, *z* = 0.35, *p* = 0.73, *R* ^2^ = 0.31).

**TABLE 3:** Multiple regression analyses testing the main and interactive effects of SM identity and LPP residuals to negative interpersonal images on depressive symptoms.

Variable	*β*	*b*	*SE*	*p*	z
SM identity	0.46	15.60	2.33	0.00	6.68
LPP residuals to negative stimuli	0.07	0.27	0.33	0.41	0.82
SM identity × LPP residuals to negative stimuli	−0.19	−1.73	0.77	0.02	−2.25

*Note: R* ^2^ = 0.28. FIML was used to account for missing data.

Abbreviations: LPP, late positive potential; SM, sexual minority.

## 4 Discussion

The current study is among the first to include both psychosocial factors and neural processes to examine disparities in depressive symptoms between SM and heterosexual adolescents. Consistent with prior literature, we found that SM adolescents reported a higher level of depressive symptoms compared with their heterosexual peers (Lucassen et al. 2017; Pachankis and Clark 2025). We also found that the severity of peer and family stressors was significantly associated with depressive symptoms and that SM adolescents were exposed to more severe interviewer‐assessed acute peer stressors than heterosexual adolescents. No significant difference emerged in the severity of family stressors between SM and heterosexual adolescents. At the neural level, LPP to both positive and negative interpersonal images moderated the relationship between SM identity and depressive symptoms even after covarying the severity of recent peer stressors. The association between SM identity and depressive symptoms was the strongest for adolescents who also showed a relatively blunted LPP to positive or negative interpersonal emotion images.

The current study offers unique insight into individual differences in emotion‐related neural processes that moderate the association between sexual orientation and depressive symptoms in youth. Specifically, LPP to interpersonal emotion stimuli might serve as a unique risk or resilience factor. Although LPP magnitudes were not directly associated with sexual orientation, a blunted LPP to both negatively and positively valenced interpersonal stimuli potentiated the association between sexual orientation and depressive symptoms in adolescents. This suggests that reduced neural responses to social stimuli in general, regardless of valence, might serve as a vulnerability for depression among SM adolescents. This finding is consistent with the ECI theory that characterizes depression as blunted emotional reactivity to both valences (Bylsma 2021) and is also consistent with our prior findings that blunted social reward responsiveness potentiated the risk of depression in SM adolescents (Long et al. 2024). This also aligns with other evidence of interactions between stress and LPP on depressive symptoms (Dickey, Pegg, et al. 2021; Dickey, West, et al. 2021). Given that the interaction findings in this paper persisted when covarying for disparities in recent peer stress exposure, it did not seem to be fully driven by recent acute peer stress, at least as measured by the Life Stress Interview (Hammen 1991) used in this study. This finding expanded the previous literature with a focus on neural reward systems (Eckstrand, Lenniger, et al. 2022; Eckstrand, Silk, et al. 2022; Forbes et al. 2021; Long et al. 2024; Seah et al. 2025) by examining neural emotional reactivity to both positive and negative interpersonal images, finding that individual differences in response to both seem to impact risk and resilience for depressive symptom in SM adolescents.

Overall, the findings point to LPP, a neurophysiological measure of emotional reactivity, as a unique neural marker that may be relevant to the emergence of depressive symptoms in SM youth. Further, this work provided empirical evidence of the interactions among multilevel risk factors, including minority stress exposure, interpersonal stressors, and individual differences in neural functions, impacting adolescent depression (Eckstrand, Lenniger, et al. 2022). In line with a developmentally informed minority stress model, increased depressive symptoms in SM adolescents with blunted neural responses to emotional stimuli are likely the result of discrimination and stigmatization against SM adolescents (Goldbach et al. 2014; I. H. Meyer 2003), rather than any inherent aspect of the identity itself. Indeed, repeated exposure to minority stress in early life—such as through parental rejection and peer victimization based on SM status—may potentiate blunted neural responses to social stimuli that can then drive depressive symptoms among SM adolescents (Hatzenbuehler 2009). Our findings warrant future research integrating comprehensive assessments of minority stress experiences in addition to measures of neural reactivity to empirically examine these associations (Goldbach et al. 2014; Meyer 2003).

Our results should be evaluated in light of a few limitations. First, we focused on depressive symptoms rather than other forms of psychopathology that may be also common in SM relative to heterosexual youth (Eckstrand, Lenniger, et al. 2022). The findings were generally consistent with prior research that characterized depression as reduced emotional reactivity across valences (Bylsma 2021; Rottenberg et al. 2005) and with prior evidence showing that reduced LPP magnitudes to rewarding or threatening stimuli are more distinct in individuals with depression (Dickey, Pegg, et al. 2021). Still, effects may not generalize to other symptoms like social anxiety, where heightened LPP responses to negative stimuli are more commonly found (Dickey, Pegg, et al. 2021). Given that sexual orientation disparities in adolescent mental health outcomes could include multiple forms of psychopathology, future studies should examine symptoms of other disorders that are prevalent among SM adolescents (Goldbach et al. 2014). Next, we only examined recent interpersonal stressors that had occurred within the past 6 months. Although results indicated that interactions between SM identity and LPP magnitude on depressive symptoms persisted when accounting for disparities in recent peer stress exposure, it is unclear whether similar patterns would be observed when considering stress exposure earlier in development and across a broader range of domains and experiences.

Furthermore, although this study focuses on interpersonal stressors, it is important to recognize the importance of other interpersonal factors that may promote resilience, such as parental acceptance and social support (Clark et al. 2022; Roe 2017). Our study did not include a measure for adult support; yet, it is an important direction for future research, such as exploring the interaction between neurophysiological markers of depression and interpersonal resources on SM adolescents’ mental health. Another limitation of the present study is that a dimensional measure of sexual identities was not included, which may obscure potential variability among specific SM identities. Our dichotomization of SM identities included “post‐gay” labels, such as pansexual, as well as capturing the identities that are still in “questioning” (Russell et al. 2009), but more nuanced, dimensional measures could yield additional insights. Although direct measures of minority stress were not included in the present study, ample research supports the developmentally informed minority stress model in which the sexual identity process itself already serves as a minority stressor during adolescence (Goldbach and Gibbs 2017). In addition, given that the current study is a secondary data analysis and the original study did not specifically recruit SM adolescents but rather focused on a wide range of symptomology across adolescents, the sample was imbalanced between heterosexual and SM adolescents, and more targeted recruitment efforts are needed in future studies. Our study is also limited by its cross‐sectional design, which does not allow for the exploration of the role of LPP in moderating changes in symptoms over time. By adopting a longitudinal design, future studies can examine whether individual differences in LPP, in combination with minority stress experience, might reflect vulnerability for later symptom development in adolescents.

Lastly, there are some potential confounds that could have impacted the overall LPP response to emotional compared to neutral images. First, the emotional image sets included human interactions, whereas the neutral images primarily featured inanimate scenes such as landscapes, city scenes, and plants. Furthermore, we did not control for stimulus properties such as luminance and image complexity. Emotional images involving people tend to be inherently more complex than neutral images without human content, as the emotional arousal level influences people's subjective ratings of complexity (Madan et al. 2018). A prior study found that LPP to emotional images was larger than LPP to neutral images, regardless of the visual complexity of the images, but LPP was larger when viewing simpler emotional images than more complex emotional images (Bradley et al. 2007). Other low‐level visual features, such as luminance, also seem to impact LPP amplitude, but LPP is modulated by emotion regardless of luminance (Jennings and Martinovic 2015). Future studies should aim to select neutral images that better match the emotional images in terms of visual complexity and luminance.

## 5 Conclusion

Overall, our study integrated multiple methods, including clinical interviews, self‐reports, and neurophysiological measurements, to evaluate interpersonal factors in the link between SM identity and depressive symptoms in adolescents. This is among the first studies to examine neural moderators of the sexual orientation disparities in adolescent depression while accounting for interview‐assessed severity of recent interpersonal stressors. Lastly, the findings also expanded the existing literature on SM identity and neural reward systems (Clark et al. 2023; Eckstrand, Lenniger, et al. 2022; Forbes et al. 2021; Long et al. 2024) to encompass neural reactivity to both positive and negative emotional valences within interpersonal contexts.

## Conflicts of Interest

The authors declare no conflicts of interest.

## Supporting information




**Supplementary Materials**: dev70092‐sup‐0001‐SuppMat.docx

## Data Availability

De‐identified data and scripts are available upon request to the corresponding author.

## Endnotes

1 We conducted primary analyses excluding those who indicated “preferred not to say” for sexual orientation and found that the interaction between SM identity and LPP residuals to negative interpersonal images remained significant (*β* = −0.23, *p* = 0.01, *z* = −2.54, *R* ^2^ = 0.27), but the interaction with LPP residuals to positive interpersonal images was no longer significant (*β* = −0.11, *p* = 0.23, *z* = −1.21, *R* ^2^ = 0.23).

2 A mixed ANOVA revealed no significant main effect of OC method (facial EOGs vs. alternative method) on LPP magnitude (*p* = 0.58) and no significant interaction between OC method and image type on LPP magnitude (*p* = 0.44). We added the dichotomous variables of whether EOGs were replaced and whether the participant used 16 or 32 channels into the models as covariates, and all primary results remained significant (*p*s < 0.05).

3 O1, O2, Oz, and Pz were used to approximate an occipital–parietal LPP and were collected for all participants regardless of the number of electrodes (i.e., 16‐ or 32‐electrode montages).
