# Supplementary material for: Interpersonal Contributors to Depression in Sexual Minority Adolescents: An Examination of Exposure to Acute Stressors and Neural Reactivity to Interpersonal Emotional Images
Source: Dev Psychobiol. 2025 Dec 2;68(1):e70092. doi: 10.1002/dev.70092 (PMC12673233; doi:10.1002/dev.70092)
Supplement: Supplementary file 1 — Supplementary Materials: dev70092‐sup‐0001‐SuppMat.docx [file DEV-68-e70092-s001.docx]

Supplementary Materials

We conducted the Shapiro-Wilk test on the dependent variable, depressive symptoms, to examine the normality assumption in the residuals and found that the assumption was violated (*W* = .94, *p* <.001). This was expected given that most participants reported low levels of depression, given the nature of psychopathology. We then used maximum likelihood with robust standard errors (MLR) to address the non-issue of the non-normal residual distribution (Mansournia et al., 2021). As a result, we found similar significant interaction effects for both LPP to positive (*β* = -.19, *p* = .041) and negative interpersonal emotional stimuli (*β* = -.19, *p* = .04). In addition, we also tested for multicollinearity in each model using the variance inflation factor (VIF) value. We found that all VIF values were below 2, indicating minimal concerns for multicollinearity (Kim, 2019).

Reference

Kim, J. H. (2019). Multicollinearity and misleading statistical results. *Korean Journal of Anesthesiology*, *72*(6), 558–569. https://doi.org/10.4097/kja.19087

Mansournia, M. A., Nazemipour, M., Naimi, A. I., Collins, G. S., & Campbell, M. J. (2021). Reflection on modern methods: Demystifying robust standard errors for epidemiologists. *International Journal of Epidemiology*, *50*(1), 346–351. https://doi.org/10.1093/ije/dyaa260
